# Association between the mental domain of the comprehensive geriatric assessment and prolonged length of stay in hospitalized older adults with mild to moderate frailty

**DOI:** 10.3389/fmed.2023.1191940

**Published:** 2023-06-23

**Authors:** Yung-Chen Yu, Chien-Chou Su, Deng-Chi Yang

**Affiliations:** ^1^Department of Nursing, National Cheng Kung University Hospital, College of Medicine, National Cheng Kung University, Tainan, Taiwan; ^2^Clinical Innovation and Research Center, National Cheng Kung University Hospital, College of Medicine, National Cheng Kung University, Tainan, Taiwan; ^3^Department of Geriatrics and Gerontology, National Cheng Kung University Hospital, College of Medicine, National Cheng Kung University, Tainan, Taiwan; ^4^School of Medicine, College of Medicine, National Cheng Kung University, Tainan, Taiwan

**Keywords:** older adults, frailty, mental domain, comprehensive geriatric assessment, prolonged length of stay

## Abstract

**Introduction:**

Previous researches have shown the risk factors of prolonged length of stay (PLOS) in hospitalized older adults, but it is unclear what are the risk factors of PLOS in hospitalized older adults with mild to moderate frailty.

**Objective:**

To identify the risk factors of PLOS in hospitalized older adults with mild to moderate frailty.

**Methods:**

We recruited adults aged ≥65 years old with mild to moderate frailty admitted to a tertiary medical center in the southern Taiwan from June 2018 to September 2018. Each individual underwent a structural questionnaire interview within 72 h after admission and 72 h after discharge. The data were collected face-to-face, including demographic characteristics, comorbidities, length of stay (LOS), and multiple domains of the comprehensive geriatric assessment. The main outcome was PLOS.

**Results:**

Individuals who had two or more drugs, were female, did not have cognitive impairment and had a Geriatric Depression Scale score ≥ 1 had a higher risk of PLOS (probability = 0.81), and these individuals accounted for 29% of the overall study population. Among male individuals younger than 87 years old, those with cognitive impairment had a higher risk of PLOS (probability = 0.76), and among male individuals without cognitive impairment, living alone was associated with a higher risk of PLOS (probability = 0.88).

**Conclusion:**

Early detection and management of mood and cognition in older adults, together with comprehensive discharge planning and transition care, may be an important part of reducing LOS in hospitalized older adults with mild to moderate frailty.

## 1. Introduction

Due to the aging population, hospitals have had to address a continuous increase in the number of older adults, most of whom present with severe illness, multimorbidity, and frailty (1). Frailty is a condition, which can lead to a reduction in the physiological reserve, cumulative functional decline, and poor responses to stressors (2, 3). It is common in older adults with acute unplanned hospitalization, and its prevalence is 2–4 times higher in older adults with acute hospitalization than those in the community (4). It is also predictive of worse clinical outcomes, including prolonged length of stay (PLOS), discharge to a destination other than home, and mortality (4). The need for intensified medical care in older frail adults, as reflected by PLOS (5), contributes to the care burden among families and contributes to the economic burden among societies.

Previous studies have shown that risk factors of PLOS include individuals at risk for functional decline (5, 6), dependence in activities of daily living (5), dependence in instrumental activities of daily living (7), acute confusion state (8), cognition impairment (5, 6), malnutrition (7), falling history (7), the number of comorbidities (5), and frailty (8). However, the risk factors of PLOS in hospitalized older frail adults remain unclear.

The early detection of older frail adults at risk for adverse hospital outcomes would help the interdisciplinary team to provide a better management plan. For this goal, a complete assessment at hospital admission may be necessary; this kind of assessment in older frail adults is called a comprehensive geriatric assessment (CGA), and it is used to establish treatment strategies and interventions in frail older adults (9). CGA was first developed in the United Kingdom, and its concepts, indications, and applications evolved over time (10). It is a multidomain, multidisciplinary diagnostic and therapeutic process performed to assess the medical, mental, social, and functional problems of older adults, thereby contributing to a tailor-made and integrated plan for management and follow-up (11).

Therefore, we conducted a CGA-based prospective cohort study to identify the risk factors of PLOS in hospitalized older adults with mild to moderate frailty.

## 2. Methods

### 2.1. Participants

We recruited adults aged ≥65 years old with mild to moderate frailty who were admitted to medical wards in a tertiary medical center from June 2018 to September 2018. The exclusion criteria were delirium, critical or terminal illness, inability to communicate, and long-term bedridden state. Informed consent was obtained from all individuals, and the study was approved by the National Cheng Kung University Hospital Institutional Review Board (A-ER-106-261). Each individual underwent a structural questionnaire interview within 72 h after admission and 72 h after discharge. The data were collected face-to-face, including demographic characteristics, comorbidities, length of stay (LOS), and multiple domains of the CGA.

### 2.2. Comprehensive geriatric assessment

The CGA involves medical, mental, social, and functional domain assessment and management. The medical domain includes malnutrition, urine incontinence, falls, and the number of drugs; the mental domain includes mood and cognition; the social domain includes educational level, marital status, and living condition; and the functional domain includes frailty evaluation. All the components of CGA mentioned above were collected within 72 h after admission. These factors were selected because they are important in older adults, and previous reports have shown an association between these factors and PLOS (5–8). Frailty was assessed by the Clinical Frailty Scale (CFS) (12). The CFS is a simple and intuitive clinical examination with a nine-point scale, and it categorizes overall performance from very fit (CFS = 1), fit (CFS = 2), managing well (CFS = 3), living with very mild frailty (CFS = 4), living with mild frailty (CFS = 5), living with moderate frailty (CFS = 6), living with severe frailty (CFS = 7), living with very severe frailty (CFS = 8), and to terminally ill (CFS = 9). Older adults with CFS = 4–6 were classified to mild to moderate frailty within 72 h after admission. Mood state was evaluated by the Geriatric Depression Scale-5 (GDS-5), which is a five-item screening tool developed in 1999 (13). The GDS scores ranged from 0 to 5, with a sensitivity of 0.97 and a specificity of 0.85 using ≥2 as a cutoff point to define the presence of depressive symptoms (13). Cognition was measured by the Short Portable Mental Status Questionnaire (SPMSQ), which is 10-item screening tool developed in 1975 (14). Incorrect answers on more than two questions indicated impaired cognitive function. One more incorrect answer was allowed for the participants with a grade school education or lower, and one less incorrect answer was allowed for the participants with a high school education or higher (14). Malnutrition was assessed by the Malnutrition Universal Screening Tool (MUST) (15), which was developed by the British Association for Parenteral and Enteral Nutrition (BAPEN). It is a screening tool to categorize adults into low risk (MUST score = 0), medium risk (MUST score = 1), and high-risk categories for malnutrition (MUST score ≥ 2) (15). The participants with MUST scores ≥2 were defined as having malnutrition in the current study. Urine incontinence was defined if the individuals reported leakage of urine ≥ 6 days within the past year. A fall was indicated if fall episodes occurred ≥2 times in the past 6 months before admission. The number of drugs was reported by the individuals themselves.

### 2.3. Comorbidity

We reviewed the electronic medical records to collect data on comorbid conditions, and the Charlson Comorbidity Index (CCI) (16) was calculated. The CCI was developed in 1987, and it is often considered the gold-standard tool for assessing comorbidities in clinical research.

### 2.4. Demographic characteristics

The demographic characteristics included age, gender, marital status, education level, economic status, and institutionalization. Marital state was categorized into married or living with a partner and living without a partner (separated, divorced, widowed, or never married) (17). Educational level was categorized into illiteracy and literacy.

### 2.5. Prolonged length of stay

Prolonged length of stay is defined as a LOS greater than the 90th percentile of hospital days (18). In the current study, PLOS was defined as a LOS longer than 20 days. In contrast to PLOS, a LOS less than 20 days was defined as non-PLOS.

### 2.6. Statistical analysis

Descriptive statistics were used to summarize the baseline characteristics in the study cohort. The continuous variables were described by medians with 25 and 75th percentiles, and the categorical variables were described by numbers and proportions. The Wilcoxon rank sum test and Pearson’s chi-squared test were used to evaluate the differences in continuous and categorical variables, respectively, in individuals with PLOS and without PLOS.

The sample sizes of the PLOS group and non-PLOS group were imbalanced, which could influence the performance of a model with respect to accuracy and bias. To address imbalanced data, the synthetic minority oversampling technique (SMOTE) (19) was used to reduce the bias in this study. SMOTE is an oversampling technique that allows researchers to generate synthetic samples for minority patients (patients with PLOS). SMOTE is based on the k-nearest neighbor algorithm and works by selecting samples that are close in the feature space, thereby generating a regression line between the samples in feature space and synthesizing a new sample at a point along with the regression line. Table 1 shows the distribution of baseline characteristics with SMOTE, and they were highly similar to the distribution of characteristics without SMOTE (Supplementary Table S1). This result indicated that SMOTE would not distort the real distribution of baseline characteristics in the study cohort. The classification and regression tree (CART), a decision tree algorithm, was used to investigate the association between baseline characteristics (features) and PLOS in older adults. CART is a hierarchical structure consisting of branches and nodes. The leaf is the end of the branch, which indicates the probability of the outcome of interest in the final set of decision rules of the tree. The Gini index is a criterion for optimal splitting of nodes in developing a decision tree model. To avoid overfitting the data, we tuned the complexity parameter (CP) to make the relative error of the decision tree smaller. Supplementary Figure S1 shows the CP for the size of the decision tree and indicates that 10 nodes were optimal.

**Table 1:** The baseline characteristics of the study population using the synthetic minority oversampling technique (SMOTE).

Characteristic	Non-PLOS, *N* = 144^*^	PLOS, *N* = 144^*^	*p* value^**^
Age	78 (72; 85)	76 (70; 82)	0.019
Male	83 (58%)	43 (30%)	<0.001
Marital status			0.3
Living without a partner	48 (33%)	57 (40%)	
Married or living with a partner	96 (67%)	87 (60%)	
Education			0.8
Illiteracy	29 (20%)	27 (19%)	
Literacy	115 (80%)	117 (81%)	
Fall			0.4
<2 falls	119 (83%)	124 (86%)	
≥2 falls	25 (17%)	20 (14%)	
Urine incontinence			0.063
No	112 (78%)	98 (68%)	
Yes	32 (22%)	46 (32%)	
Malnutrition universal screening tool	0 (0; 0)	0 (0; 1)	0.027
Number of medication use	2 (1; 4)	3 (2; 4)	0.002
Clinical frailty scale	3 (2; 5)	3 (2; 5)	0.7
Geriatric depression scale	1 (0; 2)	1 (1; 3)	0.002
Cognitive impairment			0.3
No	108 (75%)	116 (81%)	
Yes	36 (25%)	28 (19%)	
Charlson comorbidity index (CCI)	2 (1; 3)	2 (1; 3)	0.3

PLOS, prolonged length of stay; Non-PLOS: Non-prolonged length of stay.

* Median (P25; P75); *n* (%).

** Wilcoxon rank sum test; Pearson’s Chi-squared test.

The entire sample was randomly split into a 70% training set containing 202 patients and a 30% testing set containing 86 patients. The training set was used to construct models, and the testing set was used to evaluate the generalizability of models independently. The model performance was evaluated in terms of accuracy, receiver operating characteristic (ROC) curves, area under the curve (AUC), sensitivity, specificity, and predictive positive value (PPV). Statistical analyses were carried out using R (version 4.2.1) software. “rpart,” “rpart.plot,” and “pROC” were used in the current study.

## 3. Results

Two hundred eighty-eight older adults were recruited in the study cohort by using SMOTE. There were 144 individuals with PLOS and 144 individuals with non-PLOS. The mean age was 76 years old in individuals with PLOS and 78 years old in individuals with non-PLOS. The proportion of male individuals with PLOS (30%) was significantly lower than that of male individuals with non-PLOS (58%). The number of drugs reported among individuals with PLOS was higher than that in individuals without PLOS. Detailed baseline characteristics are presented in Table 1.

The performance in the training set was used to evaluate the internal prediction capacity for constructing the decision tree model. The accuracy was 0.81 (0.76, 0.87), the area under the receiver operating curve (AUROC) was 0.86 (0.81, 0.92), the sensitivity was 0.85 (0.79, 0.90), the specificity was 0.77 (0.72, 0.83), and the positive predictive value (PPV) was 0.80 (0.75, 0.86). The performance in the testing set was used to evaluate generalization capacity. The accuracy was 0.73 (0.64, 0.83), the AUROC was 0.78 (0.69, 0.87), the sensitivity was 0.82 (0.73, 0.91), the specificity was 0.66 (0.57, 0.75), and the PPV was 0.67 (0.57, 0.76). Overall, the performance in the testing set was lower than that in the training set (Table 2).

**Table 2:** The performance of classification and regression tree (CART) for classification of prolonged length of stay in training and testing datasets.

Sample performance	Training data (*n* = 202)	Testing data (*n* = 86)
	Estimate (95% CI)	Estimate (95% CI)
Accuracy	0.81 (0.76, 0.87)	0.73 (0.64, 0.83)
AUROC	0.86 (0.81, 0.92)	0.78 (0.69, 0.87)
Sensitivity	0.85 (0.79, 0.90)	0.82 (0.73, 0.91)
Specificity	0.77 (0.72, 0.83)	0.66 (0.57, 0.75)
Predictive positive value (PPV)	0.80 (0.75, 0.86)	0.67 (0.57, 0.76)

AUROC, area under the receiver operating curve.

**Figure 1:**
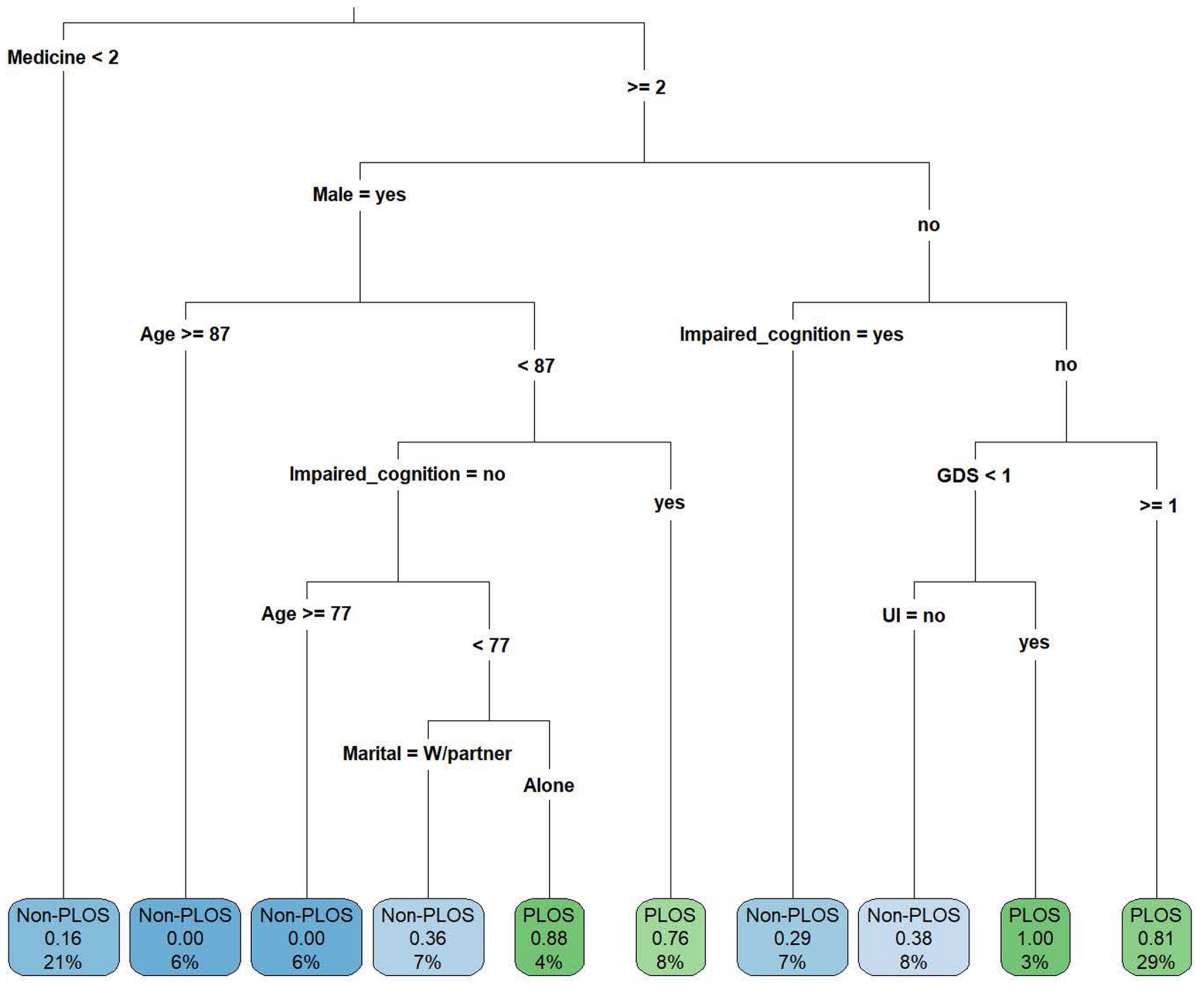
The classification and regression tree (CART) showing the decision criteria for predicting prolonged length of stay. GDS, geriatric depression scale; PLOS, prolonged length of stay; Non-PLOS, Non-prolonged length of stay; UI, urine incontinence; and W/partner, with partner.

Figure 1 indicates that if an individual reported using two or more drugs, was female, did not have cognitive impairment and had GDS ≥ 1, he or she had a higher risk of PLOS (probability = 0.81), and these individuals accounted for 29% of the overall study population. Among the male individuals, those aged less than 87 years old and with cognitive impairment had a higher risk of PLOS (probability = 0.76). Although there were two situations [P(y = PLOS|drugs used ≧ 2, female, without cognitive impairment, GDS < 1, urine incontinence) = 1.0, and P(y = PLOS|drugs used ≧ 2, male, age < 87, without cognitive impairment, live alone) = 0.88] that were associated with a higher risk of PLOS, the sample sizes were small in the leaves (3 and 4%).

## 4. Discussion

The current study demonstrated that two items in the mental domain and one item in the social domain of the CGA are associated with the PLOS in hospitalized older adults with mild to moderate frailty. If an individual reported using two or more drugs, was female, had no cognitive impairment and had GDS ≥ 1, she had a higher risk of PLOS (probability = 0.81), and these individuals accounted for 29% of the overall study population. Among male individuals aged less than 87 years old, those with cognitive impairment had a higher risk of PLOS (probability = 0.76), and among male individuals without cognitive impairment, living alone was associated with a higher risk of PLOS (probability = 0.88).

We showed that older adults with depressive symptoms were associated with a higher risk of PLOS, compatible with previous studies (20–23). GDS ≥ 2 as a cut-off point to define depressive symptoms was based on a sample from the general population (13). However, our CART results showed a different split node of GDS ≥ 1 based on female individuals using multiple medications. This split node implied that these individuals were sensitive to the risk of PLOS with mild depressive symptoms. In addition, a validation study in Taiwan (24) suggested that the cut-off point could be further lowered to 0/1 to increase the sensitivity to 100% at the screening stage.

Previous studies reported that older adults with depressive disorders had higher risks of PLOS, and those with depressive disorders showed 2-fold odds compared to those with non-depressive disorders (20–23). In addition, our results indicated that older females with depressive symptoms had higher risks of PLOS, consistent with a previous report showing that females were at risk for mental disorders compared to males (25). The sex differences in mental health could be explained by biological factors (e.g., gene and sex hormones), psychological factors (e.g., intrapersonal and interpersonal traits), and environmental factors (e.g., early life severe adversity) (26).

Depression is a common psychiatric disorder in older adults and is associated with higher complications and dependence (27). Depression in older adults is not normal aging (28), and late-life depression is characterized by atypical presentation of symptoms, including more somatic symptoms than mood symptoms, making it difficult to detect and treat (27). The association between depressed mood and PLOS in older adults could be explained by higher physical comorbidity (21), functional dependence (21), the direct impact of depressive symptoms on the immune system (29), the sympathetic nervous system (29), and the hypothalamic–pituitary–adrenal axis (29). It is also well-known that depressive individuals often do not follow medical advice for underlying medical conditions and have poorer treatment adherence (30). Older adults with depressed mood may have difficulties in effective communication with health professionals (20), and depressed mood may also affect one’s motivation toward recovery (31), both of which could result in a delay in diagnosis, treatment, and PLOS. Although it is unclear whether improving depression care will reduce LOS in acute hospitalized older adults, it could be investigated as a potential strategy to improve hospital outcomes (31).

We revealed that older adults with cognitive impairment were associated with a higher risk of PLOS, consistent with previous reports (32–36). Previous studies showed that older adults with cognitive impairment had higher risks of PLOS, and those with cognitive impairment showed a 0.8–15.3-day longer LOS than those without cognitive impairment (32–36). CART is a method that can be used for both classification and regression problems. It divides the data into subsets based on the values of the predictor variables and assigns an output value to each leaf node. In this study, we used CART to analyze the risk of PLOS in hospitalized individuals with different characteristics. We found that there were two different situations for the risk of PLOS between male and female individuals. In male individuals, cognitive impairment was a significant risk factor for PLOS, which could lead to a 0.76 probability of having PLOS, but in female individuals, cognitive impairment was not a significant risk factor for PLOS. This suggests that there are sex differences in the effect of cognitive impairment on LOS. Cognitive impairment remains a risk factor for PLOS, more so in male individuals than in female individuals.

Although we showed that older males with cognitive impairment had higher risks of PLOS, sex differences in dementia risk are unclear. One recent study suggested that females may have greater cognitive reserve but a faster decline in cognitive function than men, which could contribute to sex differences in late-life dementia (37). The difference might be due to socioeconomic, life stress or geographic factors, but further studies are needed in the future (37).

Cognitive impairment is not uncommon among older adults with acute hospitalization, but it is under recognized by health professionals with adverse outcomes (35), including disorientation (32), irritability (32), restlessness (32), falls (32, 36), decubitus ulcers (36), incontinence (32, 36), indwelling catheters (36), and medication error (36). Individuals with cognitive impairment are at a significantly greater risk of PLOS than those without cognitive impairment, possibly due to differences in effective care for individuals with cognitive impairment in hospitals and intrinsic mechanisms making these individuals at higher risk of deterioration (34). In addition, higher rates of potentially preventable events in individuals with cognitive impairment, including delirium, pneumonia, urinary tract infections, and decubitus ulcers, may also lead to PLOS (38). While individuals with dementia are mostly recognized and managed, those with cognitive impairment are mostly undetected even under routine screening (34). With comprehensive and enhanced recognition of cognitive impairment, interventions to improve care for these vulnerable groups of older adults would be possible (34).

We found that older adults living alone were associated with PLOS. One study identified caregiver stress and nursing home placement as potential modifiable risk factors for PLOS (39). Another study proposed that new formal social care requirements in survivors of acute illness and unmeasured variables of informal care requirements would be factors related to delayed discharge (40). Delays in the provision of social and therapy requirements (41) and awaiting a downstream bed (42) were also associated with PLOS in older adults. The most appropriate strategies to avoid PLOS included integrated systems between the hospitals and community care, interdisciplinary service provision, tailor-made services, and discharge planning initiated during hospitalization with regular follow-up after discharge (43).

We designed a CGA-based prospective cohort to evaluate the association between risk factors and LOS in hospitalized older adults with mild to moderate frailty. Since the approach for older adults is totally different from that for their younger counterparts, a CGA-based model, including physiological, psychological, social, and functional domain assessment and management, was used for a more thorough and detailed data collection process. We identified that the psychiatric components of geriatric syndromes, older adults with depressive symptoms, cognitive impairment, and living alone, are associated with PLOS in hospitalized older adults with mild to moderate frailty.

To our knowledge, two studies have used the CGA-based model to analyze the risk factors of PLOS in older adults. One study (5) showed that individuals at risk for functional decline, the number of comorbidities, reduced activities of daily living, cognition impairment, and signs of depression were important predictors of LOS. Another study (7) revealed that dependence in instrumental activities of daily living, malnutrition, and history of falls were associated with a longer LOS. Our results and two other studies underline the necessity of a CGA-based model for older adult care since the risk factors of PLOS belong to different domains. Although one recent systemic review and meta-analysis showed that the CGA had no significant effect on LOS, due to the presence of high heterogeneity and controversial results, the strength of evidence for the results was limited (44). Further studies are necessary to clarify the influence of the CGA-based model on LOS in hospitalized older adults with mild to moderate frailty.

Our study had several limitations. First, the number of drugs was self-reported or proxy-reported, and there could be record bias. Second, it was a single-center study with a small sample size, and the data should be interpreted with caution. Third, we only recruited older adults hospitalized with medical illness, and extrapolation of the results to other specialties should be very careful. Fourth, we used SMOTE to deal with imbalanced data, which might cause overfitting of modeling owing to synthesizing new samples with nearest neighbor selection. Thus, we tuned the complexity parameter in the decision model to reduce the bias of overfitting.

## 5. Conclusion

The mental and social domains in the CGA, including depressive symptoms, cognitive impairment, and living alone, were associated with PLOS in hospitalized older adults with mild to moderate frailty. Early detection and management of mood and cognition in older adults, together with comprehensive discharge planning and transition care, may be an important part of reducing LOS in acute hospitalized older adults with mild to moderate frailty.

## Data availability statement

The raw data supporting the conclusions of this article will be made available by the authors, without undue reservation.

## Ethics statement

The studies involving human participants were reviewed and approved by National Cheng Kung University Hospital. The patients/participants provided their written informed consent to participate in this study.

## Author contributions

D-CY and C-CS were responsible for the study design, interpretation of data, and writing, reviewing, and editing the manuscript. Y-CY was responsible for the study design, collection of data, and writing the manuscript. All authors contributed to the article and approved the submitted version.

## Funding

Funding for this study was provided by the National Cheng Kung University Hospital (Intramural grant: NCKUH-10709003). The sponsors had no involvements in the study results.

## Conflict of interest

The authors declare that the research was conducted in the absence of any commercial or financial relationships that could be construed as a potential conflict of interest.

## Publisher’s note

All claims expressed in this article are solely those of the authors and do not necessarily represent those of their affiliated organizations, or those of the publisher, the editors and the reviewers. Any product that may be evaluated in this article, or claim that may be made by its manufacturer, is not guaranteed or endorsed by the publisher.
