# Supplementary material for: Association between the mental domain of the comprehensive geriatric assessment and prolonged length of stay in hospitalized older adults with mild to moderate frailty
Source: Front Med (Lausanne). 2023 Jun 23;10:1191940. doi: 10.3389/fmed.2023.1191940 (PMC10326269; doi:10.3389/fmed.2023.1191940)
Supplement: Supplementary file 1 [file Data_Sheet_1.PDF]

Supplementary Table 1. The baseline characteristics of the study cohort without using the synthetic minority oversampling technique (SMOTE)

| Characteristic | Non-PLOS, N = 144* | PLOS, N = 16* | p value** |
|---|---|---|---|
| Age | 78 (72; 85) | 77 (70; 82) | 0.5 |
| Male | 83 (58%) | 5 (31%) | 0.044 |
| Marital status | | | 0.4 |
| Living without a partner | 48 (33%) | 7 (44%) | |
| Married or living with a partner | 96 (67%) | 9 (56%) | |
| Education | | | 0.7 |
| Illiteracy | 29 (20%) | 4 (25%) | |
| Literacy | 115 (80%) | 12 (75%) | |
| Fall | | | >0.9 |
| <2 falls | 119 (83%) | 13 (81%) | |
| ≥2 falls | 25 (17%) | 3 (19%) | |
| Urine incontinence | | | 0.5 |
| No | 112 (78%) | 11 (69%) | |
| Yes | 32 (22%) | 5 (31%) | |
| Malnutrition universal screening tool | 0 (0; 0) | 0 (0; 0) | 0.8 |
| Number of medication use | 2 (1; 4) | 3 (2; 4) | 0.2 |
| CFS | 3 (2; 5) | 3 (2; 6) | 0.6 |
| Geriatric Depression Scale | 1 (0; 2) | 1 (0; 2) | 0.4 |
| Cognitive impairment | | | >0.9 |
| No | 108 (75%) | 12 (75%) | |
| Yes | 36 (25%) | 4 (25%) | |
| Charlson Comorbidity Index (CCI) | 2 (1; 3) | 2 (1; 3) | >0.9 |

PLOS, prolonged length of stay; Non-PLOS, Non-prolonged length of stay.

*Median (P25; P75); n (%)

**Wilcoxon rank sum test; Pearson's Chi-squared test.

Supplementary Figure 1. The complexity parameter (CP) for developing the decision tree.

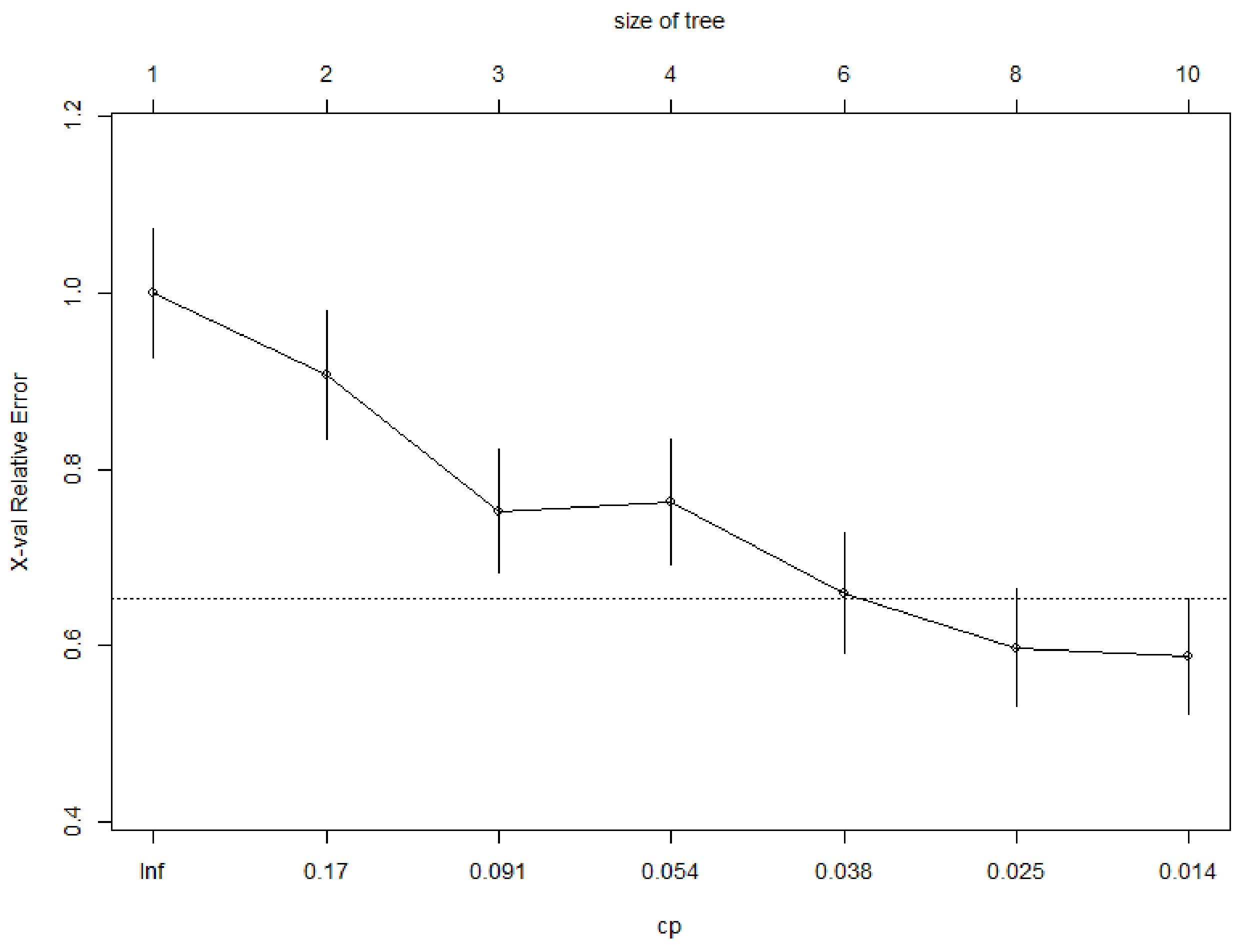


The relative error gradually tends to stabilize when the size of the tree is approximately 10.

Supplementary Figure 2. The feature importance for optimal split in decision tree.

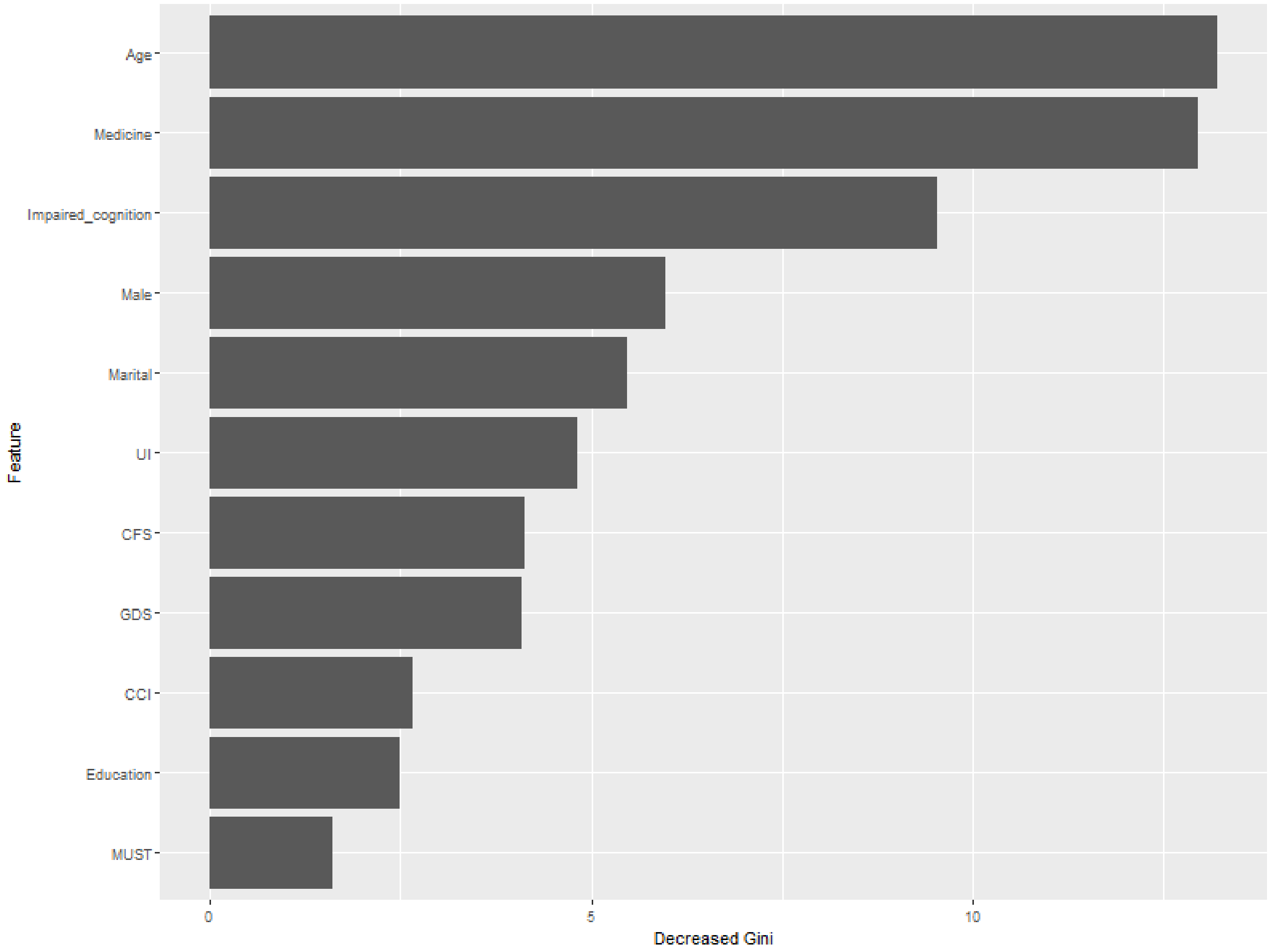


A variable's importance is the sum of the improvement in the overall Gini measure produced by the nodes in which it appears
